# Factors Influencing the Referral of Patients with Prediabetes to a Diabetes Prevention Program in the Bronx, New York

**DOI:** 10.5888/pcd20.230072

**Published:** 2023-09-21

**Authors:** Cara Stephenson-Hunter, Giovanni Pacheco, Ryung S. Kim, Qi Gao, H. Dean Hosgood, Earle C. Chambers

**Affiliations:** 1 Institute for Clinical and Translational Research KL2 Scholars Program, Albert Einstein College of Medicine, Bronx, New York; 2 Department of Family and Social Medicine, Albert Einstein College of Medicine, Bronx, New York; 3 Department of Epidemiology and Population Health, Albert Einstein College of Medicine, Bronx, New York

## Abstract

**Introduction:**

Disparate engagement in the Diabetes Prevention Program (DPP) may occur as early as the point of referral for certain subgroups, including Black and Hispanic men. We aimed to determine patient demographic and provider characteristics associated with referrals to a health system DPP in the Bronx, New York.

**Methods:**

Patient and health system characteristics for DPP-eligible patients seen in primary care between July 1, 2015, and December 31, 2017, were obtained through the electronic health record. Generalized mixed-effects modeling was used to test the association between referral rate and clinical and sociodemographic variables.

**Results:**

Of 26,727 eligible patients, 66% were female, 46% were Hispanic, and 39% were non-Hispanic Black. Only 10% (n = 2,785) of eligible patients were referred to DPP. In the adjusted analyses, lower odds of referral were observed for men versus women (OR = 0.60; 95% CI, 0.52–0.66), for non-Hispanic White versus Hispanic patients (OR = 0.53; 95% CI, 0.40–0.71), and for uninsured patients versus Medicaid patients (OR = 0.66; 95% CI, 0.54–0.80). The odds were higher for patients in the highest versus lowest hemoglobin A1c (OR = 2.49; 95% CI, 2.27–2.72) category; for those in the highest versus lowest body mass index categories (OR = 1.61; 95% CI, 1.45–1.79); for middle-aged patients (aged 45–64 y) versus those aged 18–26 y (OR =1.63; 95% CI, 1.33–2.00); and for patients being seen by a family versus an internal medicine physician (OR = 1.65; 95% CI, 1.22–2.22).

**Conclusion:**

We identified under-referral for men and highlighted other patient and health system factors associated with referral rates. Interventions to address bias in referrals and increase referrals for men at high risk for diabetes, not typically represented in DPP, are recommended.

**Summary**

**What is known on this topic?**

The Diabetes Prevention Program (DPP) has struggled to reach many at-risk patients, including Black and Hispanic men. Although the clinical encounter can be a point of entry for DPP, whether referrals contribute to disparate engagement is unknown.

**What is added by this report?**

We found that men were less likely to receive a referral to DPP, and we identified other patient and health system factors that influenced referrals to DPP.

**What are the implications for public health practice?**

The referral is an important place to intervene not only to increase the reach of this intervention but also to ensure equitable engagement for men and other populations at high risk for diabetes.

## Introduction

More than 37 million people in the US have type 2 diabetes (T2D), which is among the top 10 leading causes of death in the nation (1,2). Each year, an estimated $237 billion is spent on direct medical care and $90 billion on reduced productivity, making T2D the most expensive preventable disease in the US (1). For the more than 96 million people with prediabetes, defined as elevated hemoglobin A1c (HbA_1c_) but under the limits for T2D diagnosis, approximately 15 to 29 million will develop T2D within 3 to 5 years without intervention. Eight of 10 people with prediabetes are unaware that they have this condition, and many are not informed about actionable ways to reduce the risk of T2D (3). Health systems are in a prime position to promote T2D prevention through screening and lifestyle change interventions including through the Centers for Disease Control and Prevention’s (CDC’s) Diabetes Prevention Program (DPP). The US Preventive Services Taskforce (USPSTF) and the American Diabetes Association (ADA) recommend that providers screen for prediabetes in primary care and refer patients who have the condition to lifestyle interventions that promote healthy eating and physical activity, such as DPP (4,5). DPP remains among the most promising strategies to reduce the population burden of T2D (6,7). In clinical trials, DPP reduced the incidence of T2D among high-risk individuals with prediabetes by 58% over an average of 2.8 years, which was almost twice the 31% reduction achieved with metformin alone (6). However, despite the success of the initial trial, DPPs implemented in health system and community settings via the CDC-sponsored DPP initiative have not achieved the expected level of adoption (8–11). Furthermore, prior work has shown that DPP has struggled to reach populations with the highest T2D-related morbidity and mortality rates, such as Black and Hispanic men (12,13).

Black and Hispanic men have significantly more complications and higher death rates from T2D compared with women and men from other races and ethnicities (3). Despite these striking disparities, Black and Hispanic men are underrepresented across national DPPs (12). It is unknown whether referrals contribute to the observed disparities in DPP engagement. The earliest point of engagement is referral from a provider, usually during a routine primary care visit. A prior investigation on the reach of our Montefiore Health System (MHS) DPP (hereafter, Montefiore DPP) indicates that the proportion of men declines at each stage of program engagement and suggests that the disparity may begin at the point of referral by a health care provider (8). However, most of the literature focuses on patient priorities and barriers related to DPP engagement, while little examines health system bias in referrals (14–16). Few studies use objective referral data as opposed to provider self-report to examine referral behavior for patients at risk for T2D, and none, to our knowledge, have investigated provider referral patterns to understand patient engagement in DPP (17–20). Furthermore, previous work indicates that fewer than one-quarter of all patients referred to Montefiore DPP ever joined the program (8). The conversion rates from referral to participation to completion of the program indicate that the referral is an important place to intervene not only to increase the reach of this intervention but also to ensure equitable engagement.

The conceptual hypothesis and research questions underlying this study focus on the disparities in engagement in the Montefiore DPP among Black and Hispanic men, and the role of referrals from health care providers in contributing to these disparities. We hypothesized that the underrepresentation of Black and Hispanic men in the Montefiore DPP may begin at the point of referral, and that there may be health system bias in referrals. We also expected to find that referral patterns are influenced by patient sex, race and ethnicity, and other patient and health system factors. To address these hypotheses and research questions, we examined referral patterns for a cohort of DPP-eligible (based on HbA_1c_ and body mass index [BMI, kg/m^2^]) patients who had a primary care visit at the Montefiore health system and aimed to identify factors associated with referrals of eligible patients. The overarching goal of this study is to improve equitable access to evidence-based diabetes prevention by examining referral patterns based on patient characteristics that may warrant special attention. We hoped to identify areas where interventions can be implemented to achieve equitable access to DPPs within primary care.

## Methods

### Study population and study design

This study was approved by the MHS Albert Einstein College of Medicine Institutional Review Board. We conducted a cohort study of patients seen at a primary care clinic within MHS, between 2015 and 2017, which was before the Centers for Medicaid and Medicare’s 2018 initiative to provide reimbursement for DPP. MHS is a large, mostly urban hospital system, caring for approximately 3 million people in communities in the Bronx, Westchester, and the Hudson Valley, New York. MHS includes 11 hospitals and 19 ambulatory care sites, and its patients have almost 80% government payers.

### The Montefiore DPP

In 2015, MHS became one of the few health systems to receive CDC approval to implement a DPP (21). Before this, MHS partnered with the YMCA of Greater New York to implement DPP (10). The Montefiore DPP is implemented by the Office of Community and Population Health and offered free of charge to all eligible patients seen at one of the MHS primary care sites, including internal and family medicine, consisting of 46 clinical departments at 21 locations. As part of the implementation of the Montefiore DPP, the referral process was integrated into the Epic electronic health record (EHR) (Epic Systems Corporation). Eligible patients are referred to Montefiore DPP by health care providers, which include internists, family practitioners, and nurse practitioners, through the EHR. Montefiore DPP outreach coordinators follow up with referred patients to confirm their eligibility and facilitate enrollment in a class. Montefiore DPP is offered in English and Spanish as well as during evening hours. The Montefiore DPP implementation has been described elsewhere (21).

Patients included in this study were identified through systematic queries of the MHS EHR using the following 2015 DPP eligibility criteria: an HbA_1c_ between 5.7% and 6.4%, a BMI of 24.0 kg/m^2 ^or higher (23.0 kg/m^2^ if Asian), and having no prior diagnosis of diabetes. Exclusion criteria were pregnancy during the visit selected or a diabetes (type 1 or type 2) diagnosis based on International Classification of Diseases, 10th Edition, diagnostic codes. Patient data were extracted from the EHR using Looking Glass Clinical Analytics (Streamline Health). The cohort was collected by starting with all patients who had a referral to Montefiore DPP, then selecting a primary care visit for patients who met the DPP eligibility criteria but did not have a referral. Patients eligible for inclusion had an outpatient primary care visit during the study period. Although Montefiore DPP referrals can be made in settings other than primary care, less than 5% of all Montefiore DPP referrals originate from nonprimary care sites. The study sample included 31,524 patients eligible for Montefiore DPP between July 2015 and December 2017. Descriptions of the sample are published elsewhere (8).

### Measures

The primary outcome measure was referral to Montefiore DPP (0 = no; 1 = yes). Patient-level predictors were biological sex (male or female) and race and ethnicity as recorded in the EHR. Hispanic patients were dually included in many of the race categories since they could select “multiple races” or “none” in addition to Hispanic ethnicity. Patients were counted as Hispanic if identified as such in the EHR, regardless of race. Additionally, patients who identified more than 1 race and non-Hispanic ethnicity were included in the “other” category, as were those who identified as Asian, American Indian/Alaska Native, or Native Hawaiian/Pacific Islander, because few patients were in each category compared with the other race category. Measures of race (ie, White, Black, and other) and ethnicity (ie, Hispanic, non-Hispanic [NH]) were combined to create the following categories: NH White, NH Black, and NH other (22). To avoid obscuring results, patients without a documented race who were not reported as being of Hispanic ethnicity (n = 4,537) were excluded from the main analyses following preliminary analyses, which determined that no statistically significant differences existed between this group of patients and those with a documented race and ethnicity. Additionally, patients who had only specialist visits, such as radiology or laboratory, but did not have a primary care visit during the measurement period (n = 260), were excluded, as our investigation involved referrals done within primary care visits. Therefore, our final analytic sample included 26,727 patients.

Other covariates were selected on the basis of prior work that indicated their influence on referrals to DPP or lifestyle change interventions; these included the following sociodemographic factors: age at visit index date; insurance or payer; diabetes risk factors, including BMI; HbA_1c_; and the Charlson Comorbidity Index (CCI). CCI predicts 10-year survival in patients using multiple weighted comorbidities such as myocardial infarction, congestive heart failure, chronic obstructive pulmonary disease, liver disease, hemiplegia, moderate to severe chronic kidney disease, solid tumors, leukemia, lymphoma, and autoimmune deficiency syndrome (23). The rate of primary care visits for each patient was calculated by identifying their first primary visit in the EHR and calculating the number of visits between the initial visit and the visit index date by year and dividing by the number of years between the first and index date. Health system characteristics included specialty of provider at primary care visit, categorized as family or internal practitioner, and visit sites categorized as federally qualified health center (FQHC) and non-FQHC. FQHCs receive specific reimbursement under Medicare and Medicaid to provide health services to underserved populations. The National DPP identified FQHCs as ideal providers of DPP due to their high proportion of Medicaid recipients and comprehensive approach to care, which addresses primary, preventive, and public health education needs, as well as the social determinants of health (24).

### Statistical analysis

Descriptive statistics were summarized by means and standard deviations for continuous variables and counts and percentages for categorical variables. Student *t* tests were performed to compare the between-group difference for continuous variables. Pearson χ^2^ tests were used to compare patients’ sociodemographic and other characteristics (ie, race and ethnicity, sex, age, insurance or payer, number of visits, preferred language) across the racial and ethnic groups. DPP referral was compared by patient characteristics, diabetes risk factors (BMI and HbA_1c_), other comorbidities (CCI), and health system factors, using χ^2^ tests. The associations between Montefiore DPP referral and patient characteristics, diabetes risk factors, CCI, and health system factors (FQHC status and provider specialty) were assessed by using generalized mixed-effects models (GLMM) with logit link and referral as a binary response variable, facility as the random intercept to account for the correlation within facility, and each predictor as the single covariate (for unadjusted analyses). Covariates were then included in the multivariable model (for adjusted analyses). Odds ratios (ORs), 95% CIs, and *P* values were reported from the model. To assess whether the relationships between race and ethnicity, insurance or payer, and provider specialty on DPP referral outcomes were modified by sex, and interaction terms were run for each of these variables. However, interaction terms between sex and race and ethnicity, sex and insurance or payer, and sex and provider specialty were not significant at the *P* < .05 level and were therefore not included in the final model. Analyses were conducted by using SAS statistical software version 9.4 (SAS Institute, Inc).

## Results

Of our 26,727 Montefiore DPP-eligible patients, 46% were Hispanic and 39% were NH Black (Table 1). Approximately one-third (34%) of all eligible patients were men. For demographic characteristics, several differences were observed by racial and ethnic categories (Table 1). NH White patients were older than Hispanic patients, NH Black patients, and patients categorized as NH other (*P* < .001). NH Black patients had higher BMI (*P* < .001) and HbA_1c_ (*P* < .001) compared with all other racial and ethnic groups. Among all eligible patients, only 2,785 (10%) were referred to DPP. Significant differences were observed in characteristics among patients who were referred versus those who were not. Characteristics of patients eligible for DPP and their referral status are shown in the Appendix. We controlled for these potential confounders (ie, age and BMI) in the multivariable models comparing referral rates between categories of race and ethnicity and sex.

**Table 1:** Characteristics of Patients Eligible for the Montefiore Diabetes Prevention Program, by Race and Ethnicity, Bronx, New York, 2015–2017^a^

Characteristic	Hispanic (n = 12,416)	NH White (n = 1,245)	NH Black (n = 10,340)	NH other^b^ (n = 2,726)	All (n = 26,727)	*P* value^c^
**Sex**						
Male	33.6	48.3	30.5	39.5	33.7	<.001
Female	66.4	51.7	69.5	60.5	66.3	
**Age, mean (SD), y (range, 18–100)**	50.34 (15.33)	60.59 (14.73)	51.11 (15.34)	49.87 (14.89)	51.07 (15.41)	<.001
**Preferred language**						
English	62.2	92.0	98.3	87.1	80.1	<.001
Spanish	37.2	0.9	0.1	3.3	17.7	
Other	0.6	7.1	1.7	9.6	2.2	
**Diabetes risk factors**						<.001
**BMI, mean^d^ (SD)**	32.24 (6.17)	32.45 (6.74)	33.42 (6.98)	31.10 (5.63)	32.59 (6.51)	<.001
**HbA_1c_, mean (SD)**	5.96 (0.21)	5.95 (0.21)	5.98 (0.21)	5.97 (0.21)	5.97 (0.21)	<.001
**CCI^e^, mean (SD) (range, 0–18)**	0.96 (1.65)	1.52 (2.18)	1.09 (1.90)	0.74 (1.46)	1.02 (1.77)	<.001
**Insurance/payer**						
Medicaid	37.1	18.1	25.8	33.1	31.4	<.001
Medicare	19.3	38.5	19.2	15.8	19.8	
Commercial/employer	34.8	38.6	49.2	42.1	41.3	
Uninsured	8.8	4.8	5.8	9.0	7.5	
**Primary care visits/y, mean^f^ (SD)**	4.13 (3.94)	3.51 (3.43)	3.57 (3.30)	3.89 (4.11)	3.86 (3.71)	<.001

Abbreviations: BMI, body mass index; CCI, Charlson Comorbidity Index; FQHC, federally qualified health center; HbA_1c_, hemoglobin A1c; NH, non-Hispanic.

a Values are percentages unless otherwise indicated.

b NH other includes Asian, American Indian, Alaska Native, Pacific Islander, and multiracial patients.

c *P* values were generated by χ^2^ test for categorical values and analysis of variance for continuous variables.

d Includes a small number of patients with a BMI <24.0 (ie, Asian patients).

e Excludes 81 patients for whom CCI could not be calculated due to a lack of diagnostic codes found in the electronic health record. Lower scores indicate a better 10-year mortality rate.

f Number of primary care visits per year for each patient was calculated by identifying the first primary visit in the electronic health record; calculating the number of visits between the initial visit and the visit index date, by year; and dividing by the number of years between the first and index date.

After adjusting for other factors, men (vs women) had 40% reduced odds of being referred (OR = 0.60; 95% CI, 0.52–0.66) (Table 2). Among racial and ethnic groups, NH White patients (vs Hispanic patients) showed 47% reduced odds of being referred (OR = 0.53; 95% CI, 0.40–0.71). Compared with patients with a BMI between 24.0 and 29.0, those with a BMI between 30.0 and 34.9 showed 39% increased odds of being referred (OR = 1.39; 95% CI, 1.25–1.54); these odds increased to 61% for patients with a BMI of 35.0 or higher (OR = 1.61; 95% CI, 1.45–1.79). Patients with an HbA_1c_ of 6.0 or higher showed 2.5 times higher odds of being referred to Montefiore DPP than those with an HbA_1c_ of 5.7 to 5.9 (OR = 2.49; 95% CI, 2.27–2.72).

**Table 2:** Predictors of Patient Referrals to the Montefiore Diabetes Prevention Program, Bronx, New York, 2015–2017^a^

Variables	Unadjusted OR^b^ (95% CI)	*P* value	Adjusted OR^c^ (95% CI)	*P* value
**Sex**				
Female	1 [Reference]			
Male	0.57 (0.52–0.62)	<.001	0.60 (0.52–0.66)	<.001
**Race/ethnicity**				
Hispanic	1 [Reference]			
NH Black	1.02 (0.93–1.12)	.71	0.93 (0.84–1.02)	.12
NH White	0.49 (0.37–0.64)	<.001	0.53 (0.40–0.71)	<.001
NH other^d^	0.75 (0.64–0.87)	<.001	0.77 (0.65–0.91)	.002
**Body mass index^e^^,^ f**				
24.0–29.9	1 [Reference]			
30.0–34.9	1.50 (1.36–1.66)	<.001	1.39 (1.25–1.54)	<.001
≥35.0	1.86 (1.69–2.06)	<.001	1.61 (1.45–1.79)	<.001
**HbA_1c_ category^f^**				
5.7–5.9	1 [Reference]			
≥6.0	2.57 (2.36–2.80)	<.001	2.49 (2.27–2.72)	<.001
**Charlson Comorbidity Index^g^**				
<5	1 [Reference]			
≥5	0.80 (0.66–0.98)	.03	0.81 (0.66–1.00)	.51
**Age, y**				
18–26	1 [Reference]			
27–44	1.23 (1.02–1.50)	<.001	1.24 (1.01–1.53)	.04
45–64	1.75 (1.45–2.10)	.20	1.63 (1.33–2.00)	<.001
≥65	1.07 (0.87–1.31)	<.001	0.99 (0.76–1.28)	.91
**Insurance/payer**				
Medicaid	1 [Reference]			
Medicare	0.80 (0.71–0.91)	<.001	0.96 (0.81–1.14)	.65
Commercial/employer	0.95 (0.87–1.05)	.34	0.94 (0.85–1.05)	.27
Uninsured	0.66 (0.36–0.75)	<.001	0.66 (0.54–0.80)	<.001
**Primary care visit rate^h^**	1.04 (1.03–1.05)	<.001	1.05 (1.04–1.06)	<.001
**Provider specialty**				
Internal medicine	1 [Reference]			
Family medicine	1.80 (1.36–2.38)	<.001	1.65 (1.22–2.22)	.001
**Site FQHC status**				
Non-FQHC	1 [Reference]			
FQHC	1.59 (0.57–4.45)	.38	1.29 (0.46–3.63)	.63

Abbreviations: FQHC, federally qualified health center; HbA_1c_, hemoglobin A1c; NH, non-Hispanic; OR, odds ratio.

a Based on a generalized linear regression model for the probability of referral, primary care department was used as random intercept to account for within department cluster.

b Unadjusted results are from the bivariate analysis with each model having only 1 predictor.

c Adjusted results are from the model, which included all the variables in the table.

d NH Other includes Asian, American Indian, Alaska Native, Pacific Islander, and multiracial patients.

e Includes a small number of patients with a BMI <24.0 (ie, Asian patients).

f Change in odds of referral with each unit increase for continuous variables.

g Excludes 81 patients for whom CCI could not be calculated due to a lack of diagnostic codes found in the electronic health record. Lower scores indicate a better 10-year mortality rate.

h Primary care visit rate for each patient was calculated by identifying the first primary visit in the electronic health record; calculating the number of visits between the initial visit and the visit index date, by year; and dividing by the number of years between the first and index date.

Compared with patients aged 18 to 26 years, patients aged 45 to 64 years had a 63% increase in odds of referral (OR = 1.63; 95% CI, 1.33–2.00); a 24% increase in odds was seen in patients aged 27 to 44 years (OR = 1.24; 95% CI, 1.01–1.53). In contrast, being uninsured was associated with 34% reduced odds of referral compared with having Medicaid (OR = 0.66; 95% CI, 0.54–0.80). The odds of being referred were higher for patients who were seen by a family medicine versus an internal medicine provider (OR = 1.65; 95% CI, 1.22–2.22) (Table 2). For each one-time increase in average primary care visit per year, the odds for referral increased by 5% (OR = 1.05; 95% CI, 1.04–1.06).

To examine the sex-specific association between referral and patient characteristics, stratified analyses by sex were performed. We found that the ORs for referral were similar for male and female patients (Table 3). However, men aged 65 years or older (vs men aged 18–26 y) had 1.25 (95% CI, 0.73–2.16) increased odds of referral, and being seen by a family medicine specialist versus a specialist in internal medicine doubled the odds of referral for men (OR = 2.22; 95% CI, 1.28–3.85), although this difference was not as marked for women (OR = 1.53; 95% CI, 1.09–2.14). Women had a higher mean primary care visit rate (mean [SD], 3.6 [3.4]) than men (mean [SD], 3.4 [4.2]; *P* < .001). A higher percentage of men (8.2%) were uninsured compared with women (7.1%; *P* < .001). Moreover, a higher proportion of men had commercial insurance, while a greater proportion of women had Medicaid or Medicare insurance (*P* <.001). 

**Table 3:** Regression Model Predictors of Patient Referral to the Montefiore Diabetes Prevention Program, Stratified by Sex, Bronx, New York, 2015–2017^a^^,^ b

Variable	Male patients		Female patients	
	OR (95% CI)	*P* value	OR (95% CI)	*P* value
**Race/ethnicity**				
Hispanic	1 [Reference]			
NH Black	0.94 (0.76–1.15)	.52	0.92 (0.82–1.03)	.14
NH White	0.51 (0.31–0.85)	.01	0.55 (0.40–0.78)	<.001
NH other^c^	0.88 (0.65–1.19)	.40	0.72 (0.60–0.88)	.001
**Body mass index^d^^,^ e**				
24.0–29.9	1 [Reference]			
30.0–34.9	1.18 (0.96–1.46)	.12	1.47 (1.30–1.67)	<.001
≥35.0	1.53 (1.21–1.92)	<.001	1.66 (1.47–1.87)	<.001
**HbA_1c_ e**				
5.7–5.9	1 [Reference]			
≥6.0	2.68 (2.20–3.26)	<.001	2.44 (2.20–2.70)	<.001
**Age, y**				
18–26	1 [Reference]			
27–44	1.33 (0.85–2.08)	.21	1.22 (0.96–1.54)	.11
45–64	1.89 (1.23–2.91)	.004	1.55 (1.23–1.96)	<.001
≥65	1.25 (0.73–2.16)	.41	0.91 (0.68–1.22)	.52
**Insurance**				
Medicaid	1 [Reference]			
Medicare	0.93 (0.65–1.35)	.72	0.97 (0.80–1.19)	.80
Commercial	1.09 (0.88–1.36)	.42	0.90 (0.80–1.02)	.10
Uninsured	0.71 (0.48–1.05)	.08	0.64 (0.51–0.80)	<.001
**Charlson Comorbidity Index^f^**				
<5	1 [Reference]			
≥5	0.67 (0.40–1.13)	.13	0.84 (0.67–1.06)	.15
**Primary care visit rate^g^**	1.05 (1.03–1.07)	<.001	1.05 (1.03–1.06)	<.001
**Provider specialty**				
Internal medicine	1 [Reference]			
Family medicine	2.22 (1.28–3.85)	.005	1.53 (1.09–2.14)	.01
**FQHC status**				
Non-FQHC	1 [Reference]			
FQHC	1.70 (0.66–4.37)	.27	1.22 (0.41–3.60)	.72

Abbreviations: FQHC, federally qualified health center; HbA_1c_, hemoglobin A1c; NH, non-Hispanic; OR, odds ratio.

a Based on a generalized linear regression model for the probability of referral, primary care department was used as random intercept to account for within department cluster.

b Adjusted results are from the model, which included all the variables in the table.

c NH other includes Asian, American Indian, Alaska Native, Pacific Islander, and multiracial patients.

d Includes a small number of patients with a BMI <24.0 (ie, Asian patients).

e Change in odds of referral with each unit increase for continuous variables.

f Excludes 81 patients for whom CCI could not be calculated due to a lack of diagnostic codes found in the electronic health record. Lower scores indicate a better 10-year mortality rate.

g Primary care visit rate for each patient was calculated by identifying the first primary visit in the electronic health record; calculating the number of visits between the initial visit and the visit index date, by year; and dividing by the number of years between the first and index date.

## Discussion

This study aimed to understand the influence of sex, race and ethnicity, and other patient and health system factors on referrals to Montefiore DPP by examining referral patterns for a cohort of DPP-eligible patients seen for a primary care visit at MHS within a 2-year period. As predicted, after controlling for all other confounders, men had lower odds of being referred compared with women. Little is known about the reasons behind this sex-related bias in referrals. Some reasons may include providers’ beliefs that the intervention is less appropriate for or effective among men than women. However, among racial and ethnic groups, NH White patients had the lowest odds of referral to Montefiore DPP, which was true for men and women. This finding was unexpected and not observed in the literature.

As expected, increasing BMI and HbA_1c_ values corresponded to increased odds of Montefiore DPP referral. However, middle age was associated with higher odds of Montefiore DPP referral but being aged 65 years or older was not. Also, being uninsured versus having Medicaid decreased the odds of being referred. These findings align with a population-based cross-sectional analysis of 2016 and 2017 National Health Interview Survey data, which found that higher HbA_1c_ and other diabetes risk factors, middle age, and insurance were positively associated with referrals to and engagement in DPP (16). The fact that the Montefiore DPP was offered free of charge to all eligible patients should have eliminated insurance-related factors known to influence provider referrals. A higher percentage of men were uninsured compared with women. One study found that providers were less likely to refer patients with prediabetes to intensive behavioral interventions when they believed the patient lacked the resources — including time, transportation, and finances — to participate (25). Being uninsured could be associated with other economic as well as social factors, such as being unemployed or having undocumented immigrant status, that may similarly influence provider referrals to Montefiore DPP.

Although FQHC status was not a significant predictor of referrals, being seen by a family medicine provider showed increased odds of being referred. Researchers have noted several differences in training and approach to care between family and internal medicine, including family medicine specialists’ offering more lifestyle counseling compared with internists (26). We did not have the information to determine why this might be occurring; however, it could be related to training. Additionally, a significantly higher proportion of men saw family medicine providers compared with women (*P* = .005). It is important to note that 90% of patients eligible for DPP were never referred during the period observed. Although the USPSTF recommends that primary care providers refer patients with prediabetes to intensive lifestyle interventions, including DPP, prior research indicates several reasons that providers do not refer (19,25). Known barriers to provider-made referrals to DPP include a lack of linkages to referral services, knowledge of program availability, and affordability for eligible patients (17,25). However, referrals were integrated within the EHR, and MHS provided informational sessions around Montefiore DPP, including how to generate referrals.

Referrals are important to achieve enrollment and care outcomes for patients with prediabetes. Most referrals do not translate into enrollment since several patient and system factors contribute to patient nonenrollment or unenrollment following a provider referral to Montefiore DPP (8,10). Some reasons are the time between referral and available classes to enroll interested patients in, and patient disinterest or inability to participate (8,10,27). Our MHS data show that between 2015 and 2017, a total of 3,281 patients were referred to Montefiore DPP and among these patients, 747 participated in at least 1 session (8); thus, the conversion rate for referral to enrollment was 23%. This rate is lower than reported by another health system DPP, which was 41% before an EHR intervention, including a prediabetes registry and provider decision-making support, which resulted in 7 times the number of referrals and a 28% increase in referral to enrollment conversion rates in 1 year (28). Similar interventions may be explored with the MHS EHR to increase referrals and participation. 

Several limitations should be noted in interpreting our findings. Missing or misclassified race data in EHR may have potentially biased results. However, our preliminary analysis did not show significant differences in outcomes between those with missing race in the EHR and the rest of the study sample. Furthermore, the study had a small sample of NH White patients, while the NH other category was notably large and heterogeneous, encompassing multiracial, Asian, Pacific Islander, and other sparsely represented racial groups. This categorization could potentially overlook the inherent diversity among these different racial groups, which presents a challenge when investigating racial and ethnic disparities in care. However, the race and ethnicity of the sample is representative of MHS patients and residents of the Bronx, New York. Our data span 2 years and several primary care sites within 1 health system serving predominantly urban-dwelling, low-income Black and Hispanic patients. Therefore, findings may not be generalizable to other health system DPPs. Although the Montefiore DPP referral process has likely changed and adapted over time and at differing times across sites, we assume that the process, including recording of referrals in the EHR, was stable across sites during the period represented in this study. These data were captured before the 2018 Medicaid reimbursement plan and the COVID-19 pandemic, both of which led to significant changes to Montefiore DPP delivery and access, including virtual sessions. We did not measure other potential factors that influenced provider referrals, such as patient–provider interactions, including conversations about interest in DPP and ability to participate, patient income, physical limitations, and other unmeasured factors that may influence a provider’s decision to refer.

Like many DPPs serving low-income and historically minoritized communities, MHS struggles to meet enrollment and weight-loss requirements needed to retain CDC recognition (29). The costs to health systems that support these communities like Montefiore DPP, and under-reimbursement by Medicaid, present an even larger challenge to Montefiore DPP sustainability (30).

Our study’s findings indicate that the sex disparity in Montefiore DPP enrollment was likely influenced by the rate at which men were referred to the program. Further study is needed to understand providers’ perspectives on Montefiore DPP and referring eligible men. Women may engage more with the health system. We found that women had higher mean visit rates compared with men. Therefore, in addition to interventions to improve equitable point-of-care referrals, the EHR could be used to identify and refer eligible and typically under-referred patients (retrospective referral methods) to improve the reach of Montefiore DPP for high-risk groups, such as men. To increase engagement for men with prediabetes, MHS in collaboration with the New York City Department of Health and Mental Hygiene developed Power-up, a men-only DPP, that is currently being tested in a randomized trial.

## Appendix Referral to the Montefiore Diabetes Prevention Program, by Demographic, Health Status, and Visit Characteristics, Bronx New York, 2015–2017^a^

**Table:**

Characteristic	Not referred (n = 23,942)	Referred (n = 2,785)	*P* value^b^
**Sex**			
Male	8,378 (35.0)	625 (22.4)	<.001
Female	15,564 (65.0)	2,160 (77.6)	
**Race/ethnicity**			
Hispanic	10,996 (45.9)	1,420 (51.0)	<.001
NH White	1,184 (4.9)	61 (2.2)	
NH Black	9,248 (38.6)	1,092 (39.2)	
NH other^c^	2,514 (10.5)	212 (7.6)	
**Age, y**			
18–26	1,556 (6.5)	147 (5.3)	<.001
27–44	6,582 (27.5)	722 (25.9)	
45–64	10,954 (45.8)	1,528 (54.9)	
≥65	4,850 (20.3)	388 (13.9)	
**Preferred language**			
English	19,240 (80.4)	2,160 (77.6)	<.001
Spanish	4,135 (17.3)	593 (21.3)	
Other^d^	567 (2.4)	32 (1.1)	
**Body mass index**			
24.0–29.9^e^	10,177 (42.5)	864 (31.0)	<.001
30.0–34.9	7,288 (30.4)	907 (32.6)	
≥35.0	6,477 (27.1)	1,014 (36.4)	
**HbA_1c_**			
5.7–5.9	12,880 (53.8)	902 (32.4)	<.001
6.0–6.4	11,062 (46.2)	1,883 (67.6)	
**Charlson Comorbidity Index**			
<5	22,712 (94.9)	2,665 (95.7)	.08
≥5	1,153 (4.8)	116 (4.2)	
Missing	77 (0.3)	4 (0.1)	
**Insurance/payer**			
Medicaid	7,385 (30.8)	1,017 (36.5)	<.001
Medicare	4,830 (20.2)	459 (16.5)	
Commercial/employer	9,898 (41.3)	1,141 (41.0)	
Uninsured	1,829 (7.6)	168 (6.0)	
**Primary care visit rate, mean (SD)** ^f^	3.78 (3.64)	4.49 (4.13)	<.001
**Provider specialty**			
Internal medicine	18,251 (76.2)	1,881 (67.5)	<.001
Family medicine	5,691 (23.8)	904 (32.5)	
**Practice type**			
FQHC	8,300 (34.7)	1,270 (45.6)	<.001
Non-FQHC	15,642 (65.3)	1,515 (54.4)	

Abbreviations: FQHC, federally qualified health center; HbA_1c_, hemoglobin A1c; NH, non-Hispanic.

^a^ Values are no. (%) unless otherwise indicated.

^b^ *P* values were generated by χ^2^ test for categorical values and Student *t* test for continuous variables.

^c^ NH other includes Asian, American Indian, Alaska Native, Pacific Islander, and multiracial patients.

^d^ Other includes all non-English non-Spanish languages and dialects.

^e^ Includes a small number of patients with a BMI <24.0 (ie, Asian patients).

^f^ Primary care visit rate for each patient was calculated by identifying the first primary visit in the electronic health record; calculating the number of visits between the initial visit and the visit index date, by year; and dividing by the number of years between the first and index date.
